# Phenolic Antioxidants in Food: A Comparative Review of Chromatographic, Spectroscopic, and Electrochemical Detection Methods

**DOI:** 10.3390/foods15183270

**Published:** 2026-09-16

**Authors:** Yani Zhao, Qiongya Lu, Jinlan Tu, Zhouyuan Zhao, Bin Zou

**Affiliations:** School of Food Science and Engineering, Jiangsu University, No. 301 Xuefu Road, Zhenjiang 212013, China

**Keywords:** phenolic antioxidants, detection techniques, chromatographic analysis, spectroscopic analysis, electrochemical detection

## Abstract

The detection of phenolic antioxidants in food is essential for ensuring food safety. Chromatographic analysis, spectroscopic analysis, and electrochemical detection are three mainstream techniques widely applied for qualitative and quantitative analysis of antioxidants in complex matrices. However, existing reviews mostly focus on single techniques, lack systematic cross-platform comparisons. This paper reviews the classification of phenolic antioxidants, summarizes recent advances in each detection technique, and provides a horizontal comparison in terms of sensitivity, selectivity, analytical efficiency, cost, and applicable scenarios. Chromatography remains the gold standard for confirmatory analysis, spectroscopy is suitable for rapid screening, and electrochemistry shows unique potential for on-site real-time monitoring. Each technique has distinct advantages and limitations, and their integration represents a promising direction for comprehensive food safety monitoring. Future development trends, including intelligence, portability, and multi-technique integration, are also discussed to guide practical method selection for food-safety detection.

## 1. Introduction

Oxidative deterioration is a major challenge affecting food quality and shelf life, triggering off-flavour formation, nutrient loss, and generation of potentially harmful secondary products [1,2,3,4]. To prevent such deterioration, the addition of antioxidants is the most widely adopted industrial strategy [5,6,7]. Food antioxidants are generally classified into natural and synthetic categories classified by source [8,9,10]. Natural phenolic antioxidants are mainly extracted from plants, including tea polyphenols, gallic acid, proanthocyanidins, etc. Nevertheless, natural antioxidants commonly suffer drawbacks such as poor stability, limited oil solubility, high cost, and large variability in antioxidant efficacy originating from raw-material sources and extraction processes [11]. Therefore, synthetic phenolic antioxidants, due to their stable chemical properties, significant antioxidant effects, and low production costs, continue to hold a dominant position in the food and even cosmetic industries.

Despite their widespread use, the safety of synthetic phenolic antioxidants has drawn considerable regulatory and scientific scrutiny. Toxicological studies in laboratory animals have demonstrated that high-dose exposure to BHA (Butylated hydroxyanisole) is associated with hepatocellular lesions and hyperplasia, leading the International Agency for Research on Cancer (IARC) to classify BHA as a Group 2B agent (possibly carcinogenic to humans) based on sufficient evidence in experimental animals [12]. Endocrine-disrupting potential of BHA has also been suggested in in vitro studies [13]. For TBHQ (t-Butylhydroquinone), subchronic and chronic exposure studies have indicated neurotoxic potential and DNA-damaging effects at elevated doses in rodent models [13,14,15,16]. PG (Propyl gallate) has been associated with cytotoxic and organ toxic effects at high concentrations in animal studies and in vitro models [17,18]. Although the direct translation of these animal findings to human health risks at normal dietary exposure levels remains a subject of ongoing research, the precautionary principle in food-safety regulation has prompted global regulatory bodies to establish strict maximum permitted levels and acceptable daily intakes (ADIs) for these compounds [19].

Given the potential risks associated with certain synthetic antioxidants as identified in toxicological studies, food safety regulatory agencies in major economies worldwide have established clear regulations regarding their scope of use and maximum permitted levels. For instance, the “National Food Safety Standard—Use Standards of Food Additives” (GB 2760-2024) of China specifies that the maximum allowable usage amounts of BHA, BHT (butylated hydroxytoluene) and TBHQ in oils are 0.2 g/kg, while the maximum allowable concentration of PG is 0.1 g/kg [20]. The European Food Safety Authority (EFSA) has set acceptable daily intakes (ADIs) of 1.0 mg/kg bw/day for BHA, 0.25 mg/kg bw/day for BHT, 0.7 mg/kg bw/day for TBHQ, and 0.5 mg/kg bw/day for propyl gallate (PG) [21,22,23,24]. In the United States, the FDA has classified BHA and BHT as GRAS (Generally Recognized as Safe) substances, with their use subject to good manufacturing practices (GMP) [25,26]. These regulatory frameworks require reliable analytical methods for enforcement and compliance monitoring.

Although the detection of food antioxidants has received widespread attention and there are numerous review articles on the subject, a thorough review reveals that most existing reviews focus on a single aspect—such as the optimization of chromatographic methods, the modification of sensor materials, the evaluation of natural antioxidant activity, or the determination of total antioxidant capacity—and lack a systematic cross-comparison of different detection techniques. In terms of sensitivity, selectivity, analytical throughput, sample pretreatment complexity, and equipment cost, no comprehensive assessment currently exists. A unified standard for evaluating the advantages and disadvantages of these three technologies has not yet been established. Moreover, the applicability and anti-interference ability of detection methods to different food matrices also lack systematic analysis. To address this gap, this review provides a comprehensive and critical comparison of the three major detection platforms, with the aim of offering practical guidance for method selection in food safety monitoring and identifying future research directions. Therefore, this paper first introduces the classification system for phenolic antioxidants in food and the requirements for their detection. It then systematically summarizes the current status and research progress of the three mainstream detection techniques—chromatographic analysis, spectroscopic analysis, and electrochemical detection—and conducts a comparative analysis from multiple perspectives, including detection performance, scope of application, complexity of sample preparation, and application scenarios. Based on this, the advantages and limitations of the three types of technologies are analyzed. Finally, the paper looks ahead to the development trends in smart sensing, portable detection, and multi-technology integration, with the aim of providing guidance for the appropriate selection of detection methods and technological iteration in food safety monitoring.

## 2. The Development of Antioxidant Classification and Multi-Dimensional Comparison

### 2.1. Analytical Objectives of Antioxidant Detection

Before discussing specific detection techniques, it is essential to clarify that “antioxidant detection” in food analysis encompasses three conceptually distinct analytical objectives, which differ fundamentally in their methodological requirements and interpretative value.

#### 2.1.1. Quantitative Analysis of Individual Antioxidant Compounds

The determination of specific target antioxidants (e.g., BHA, TBHQ, PG) in food matrices. This objective requires chromatographic separation or highly selective sensors (e.g., molecularly imprinted electrochemical sensors) to distinguish the target from co-existing interferents. Quantification is achieved through external or internal standard calibration.

#### 2.1.2. Total Phenolic Content (TPC) Determination

The measurement of the total amount of phenolic substances in a sample, typically using the Folin–Ciocalteu colorimetric method. This approach provides a composite index reflecting the overall phenolic pool but offers no information on individual compound identities or concentrations.

#### 2.1.3. Total Antioxidant Capacity (TAC)/Activity Assessment

The evaluation of the integrated free-radical scavenging or reducing capacity of a sample using in vitro assays such as ABTS (2,2’-Azinobis (3-ethylbenzothiazoline-6-sulfonic acid)), DPPH (1,1-Diphenyl-2-picrylhydrazyl), or FRAP (Ferric Reducing Antioxidant Power). This parameter reflects the overall antioxidant performance of the sample matrix, which is particularly relevant for functional foods and natural product extracts, but does not correlate directly with the concentration of any single antioxidant compound.

These three analytical objectives are not interchangeable and require distinctly different methodological approaches. The following sections will systematically discuss the three major detection platforms—chromatography, spectroscopy, and electrochemistry—with explicit reference to which analytical objective(s) each technique can address.

### 2.2. Classification of Phenolic Antioxidants

From a chemical perspective, food antioxidants do not constitute a homogeneous group with a single structure. Instead, they are composed of numerous compounds with different sources, solubilities, and mechanisms of action. They may originate from secondary metabolism of plants, or be artificially synthesized; some are lipophilic and suitable for lipid systems, while others are hydrophilic and active in aqueous media; certain antioxidants directly terminate free-radical chain reactions via phenolic hydroxyl groups, while others exert auxiliary effects through indirect pathways such as chelating metal ions or quenching singlet oxygen [27,28,29,30,31]. Different antioxidants vary significantly in terms of efficacy, strength, safety and cost. Therefore, before discussing the detection methods, a multi-dimensional classification framework should be established to compare the advantages and disadvantages of various antioxidants (Table 1). Although the subsequent detection methodology discussion focuses primarily on phenolic antioxidants, a comprehensive classification including non-phenolic types is presented here to provide a complete context for understanding the structural and physicochemical properties that influence analytical method selection.

Antioxidants can be categorized along multiple independent dimensions, including source, solubility, and chemical structure. These dimensions are not mutually exclusive; a single antioxidant may fall into different categories across dimensions (e.g., BHA is synthetic, lipophilic, and phenolic). When categorized by source, antioxidants can be classified into natural and synthetic antioxidants. Natural antioxidants refer to antioxidant-active substances obtained from plant, microbial, or animal sources through processes such as physical extraction, enzymatic hydrolysis, or solvent extraction, and which have not undergone artificial chemical modification [32,33,34]. They are generally regarded as safer and healthier options and are widely present in daily diets. Common types include vitamins (such as vitamin C, vitamin E), carotenoids (such as lycopene, beta-carotene, astaxanthin), polyphenols (such as tea polyphenols, resveratrol in grapes, anthocyanins in berries, and isoflavones in soybeans), and rosemary extract, etc. [35,36]. In addition to directly eliminating free radicals, many natural antioxidants can also exert comprehensive health benefits by regulating the body’s own endogenous antioxidant system and anti-inflammatory pathways. However, their limitations lie in the high extraction costs and the poor stability and varying bioavailability of some components. Synthetic antioxidants are antioxidant-active substances prepared through chemical synthesis and with specifically designed molecular structures [37,38,39]. Common varieties include BHA, BHT and TBHQ, which have stable chemical properties and strong antioxidant effects, and can significantly extend the shelf life of food. However, although their use in compliance is considered safe, consumers have always had concerns about the long-term safety of consuming them. Some compounds have potential toxic risks at high doses, which has driven the food industry to continuously seek natural alternatives.

Based on solubility, antioxidants can be classified into oil-soluble and water-soluble types. Oil-soluble antioxidants are mainly soluble in oils and organic solvents, and can exert antioxidant effects in lipid environments such as cell membranes and lipoproteins [40]. Common examples include vitamin E (tocopherol), butylated hydroxyanisole (BHA), and BHT, etc. They are adept at protecting foods rich in oils from oxidative rancidity. In the human body, they mainly function to protect the lipid layer of cell membranes from free radical attacks [41]. Their key characteristic is fat-solubility, enabling accumulation within the human body; therefore, dosage intake should be carefully controlled. Water-soluble antioxidants mainly dissolve in water and function in aqueous environments such as plasma and cytoplasm. Common representatives include vitamin C, tea polyphenols, and anthocyanins, etc. [42,43]. They are mainly applied in water-based food systems. In the human body, they are responsible for eliminating free radicals in the blood and cytoplasm, and can also work in synergy with oil-soluble antioxidants [44,45]. For instance, vitamin C can regenerate the oxidized vitamin E, restoring its activity. They exhibit broad-spectrum activity yet are poorly retained in the human body and require continuous dietary supplementation.

Based on chemical structure, antioxidants can be classified into phenolic and non-phenolic types. Phenolic antioxidants are structurally defined as compounds bearing one or more phenolic hydroxyl groups (i.e., hydroxyl groups directly attached to an aromatic ring). Their primary antioxidant mechanism involves the donation of a hydrogen atom from the phenolic OH group to free radicals via hydrogen atom transfer (HAT), thereby terminating radical chain reactions while forming relatively stable phenoxyl radicals through resonance stabilization. Additionally, phenolic compounds can act through single electron transfer (SET) mechanisms, and their antioxidant activity is influenced by factors such as redox potential, pH, solvent, and concentration. Some phenolic varieties also exhibit metal-chelating capacity, indirectly inhibiting oxidation reactions [46,47,48,49]. Typical representatives include natural sources of tea polyphenols, tocopherol (vitamin E), rosmarinic acid, resveratrol, as well as synthetic substances such as BHA, BHT, and TBHQ [50,51,52]. Phenolic compounds usually possess excellent redox cycling capabilities. Some varieties can also chelate metal ions, indirectly inhibiting the occurrence of oxidation reactions. Non-phenolic antioxidants refer to substances whose structures do not contain phenolic hydroxyl groups and exert their antioxidant effects through other chemical mechanisms. Their mechanisms of action include either relying on the enol structure (such as vitamin C) to provide reducing hydrogen atoms, or by capturing reactive oxygen species or peroxide intermediate products to interrupt the oxidation process [53,54]. Typical examples include vitamin C (ascorbic acid), coenzyme Q10, carotenoids (such as lycopene and astaxanthin), and alpha-lipoic acid [55,56,57]. It is precisely because of the significant differences in the sources, solubility and chemical structure of antioxidants that the detection of antioxidants becomes even more important.

**Table 1:** A Comparison of Different Categories of Antioxidants.

Category	Representative	Source	Solubility (logP)	Structural Features	Primary Antioxidant Mechanism	Safety/Regulatory Status	Analytical Properties	Main Analytical Challenges
Synthetic monophenols	BHA	Synthetic	~3.5	2(3)-tert-butyl-4-hydroxyanisole (mixture of isomers)	HAT donor; moderate chain-breaking activity [58]	IARC Group 2B; EFSA ADI: 1.0 mg/kg bw/day	UV λmax 288 nm; electroactive; volatile [59]	Moderate UV response; co-elution with lipid matrix
Synthetic polyphenols	TBHQ	Synthetic	~2.9	2-tert-butyl-1,4-dihydroxybenzene	Strong HAT and SET donor; most effective among synthetic types [60]	EFSA ADI: 0.7 mg/kg bw/day; banned in Japan	UV λmax 290 nm; highly electroactive; thermally labile [61]	Prone to oxidation; thermal decomposition
Synthetic polyphenols	PG	Synthetic	~2.0	Propyl 3,4,5-trihydroxybenzoate	Strong HAT donor; metal chelation [62]	EFSA ADI: 0.5 mg/kg bw/day	UV λmax 272 nm; highly electroactive; polar [63]	Poor retention on conventional C18 columns
Natural tocopherols	α-, γ-, δ- tocopherol	Natural	>7	Chromanol ring with phytyl side chain; phenolic OH	HAT + singlet oxygen quenching; activity: δ > γ > α for HAT [64]	GRAS (FDA 21 CFR 182.3890)	UV λmax 292 nm; weak electroactive; fluorescence (ex 295/em 330 nm) [65]	Fluorescence requires optimization; poor thermal stability for GC
Natural tea polyphenols	EGCG	Natural	~1.5	Gallate ester with 8 phenolic OH groups	Very strong HAT/SET donor; metal chelation; pro-oxidant at high doses [66]	Generally recognized as safe; rare hepatotoxicity at high supplement doses	UV λmax 280, 325 nm; highly electroactive; non-volatile [67]	Poor stability at neutral pH/high temperature; matrix interference
Non-phenolic ascorbic acid	Ascorbic acid	Natural/synthetic	~−1.9	Enediol structure (no phenolic ring)	SET donor; regenerates tocopherol; pro-oxidant with metal ions [68]	GRAS (FDA 21 CFR 182.3013)	UV λmax 265 nm; electroactive; unstable in solution [69]	Unstable to heat, light, and oxygen
Non-phenolic carotenoids	β-carotene	Natural	~15	Polyene chain (no phenolic OH)	Singlet oxygen quenching; poor HAT donor [70]	Approved as food colorant/nutrient supplement;	UV–Vis λmax 450, 475 nm; non-electroactive; non-volatile [71]	Requires normal-phase LC; easily oxidized

## 3. Classification and Research Progress of Detection Technology

The detection methods for antioxidants in food have continuously evolved with the advancement of analytical chemistry techniques, forming a multi-level technical system ranging from classical chemical analysis to modern instrumental analysis and to on-site rapid detection. According to the different detection principles, the existing methods can be classified into three major categories: chromatographic analysis, spectroscopic analysis, and electrochemical detection. Among these, chromatography has become the gold standard for confirmatory analysis in laboratories due to its exceptional separation capabilities and quantitative accuracy; spectroscopic techniques are well-suited for high-throughput screening because of their ease of operation and rapid response; and electrochemical detection has garnered significant attention in real-time on-site monitoring due to its high sensitivity and great potential for miniaturization. Each of these three types of technology has its own strengths, and they complement one another in various application scenarios. The following section first outlines the overall development trajectory of detection technologies, followed by a systematic discussion of research progress for each category.

### 3.1. Development Process of Detection Technology

As the food matrix components become increasingly complex, food safety regulatory standards become stricter, and the actual demand for on-site rapid quality control in the food industry keeps rising, the food antioxidant detection technology has undergone multiple technological updates. The antioxidant detection technology can be divided into three stages.

### 3.2. Simple Instrument Analysis Stage in the Laboratory

During this period, traditional analytical methods such as chemical titration and spectrophotometry were mainly relied upon. For instance, iodometric methods based on the reductivity of antioxidants, determination of iron reductivity, and colorimetric quantitative analysis using color reactions (such as the color reaction between BHA and 2,6-dichloroquinone-4-chloroimide) were employed. These methods required simple instruments and were suitable for routine laboratory analysis, but had limited sensitivity and were susceptible to interference from complex food matrices, making them unable to meet the requirements for precise quantification and simultaneous detection of multiple components. This stage mainly focused on qualitative analysis and rough quantification.

### 3.3. Precision Instrument Analysis Stage

With the widespread use of sophisticated analytical instruments and the gradual improvement of chromatographic theory, the detection methods for antioxidants have evolved from simple qualitative testing to precise quantitative testing, and the detection accuracy has also increased significantly. Representative techniques include high-performance liquid chromatography (HPLC), gas chromatography (GC), liquid chromatography tandem mass spectrometry (LC-MS/MS), and gas chromatography-tandem mass spectrometry (GC-MS/MS) [72,73,74,75,76]. These techniques rely on chromatographic separation to achieve component splitting, combined with mass spectrometry and ultraviolet detectors to complete precise quantification. They feature excellent detection repeatability, low quantification error, and significantly improved sensitivity compared to traditional chemical methods. For example, Xu et al. [77] developed a new method combining dispersed liquid–liquid microextraction with high-performance liquid chromatography for the simultaneous determination of six synthetic phenolic antioxidant compounds in edible oil. By optimizing parameters such as extraction solvent, dispersion solvent, and ultrasonic time, this method requires only 25 mg of sample and 1 mL of organic solvent, and can complete the sample preparation in approximately 5 min. Method validation demonstrated that each target compound had good linearity within the range of 0.1–500 mg/L, with spiked recovery rates ranging from 86.3% to 102.5%, and RSD (relative standard deviation) values below 3.5%. The results of actual sample determination showed that TBHQ was the most frequently detected component, and none of the analyzed samples exceeded the regulatory limit. This method is simple, rapid, and requires only minimal organic solvent consumption, providing an effective tool for the quality control of antioxidants in edible oil. The introduction of chromatographic instrument analysis technology has effectively made up for the shortcomings of early detection methods in terms of accuracy and specificity, laying a methodological foundation for the precise detection of phenolic antioxidant residues in food.

### 3.4. Rapid Detection Stage

**Figure 1:**
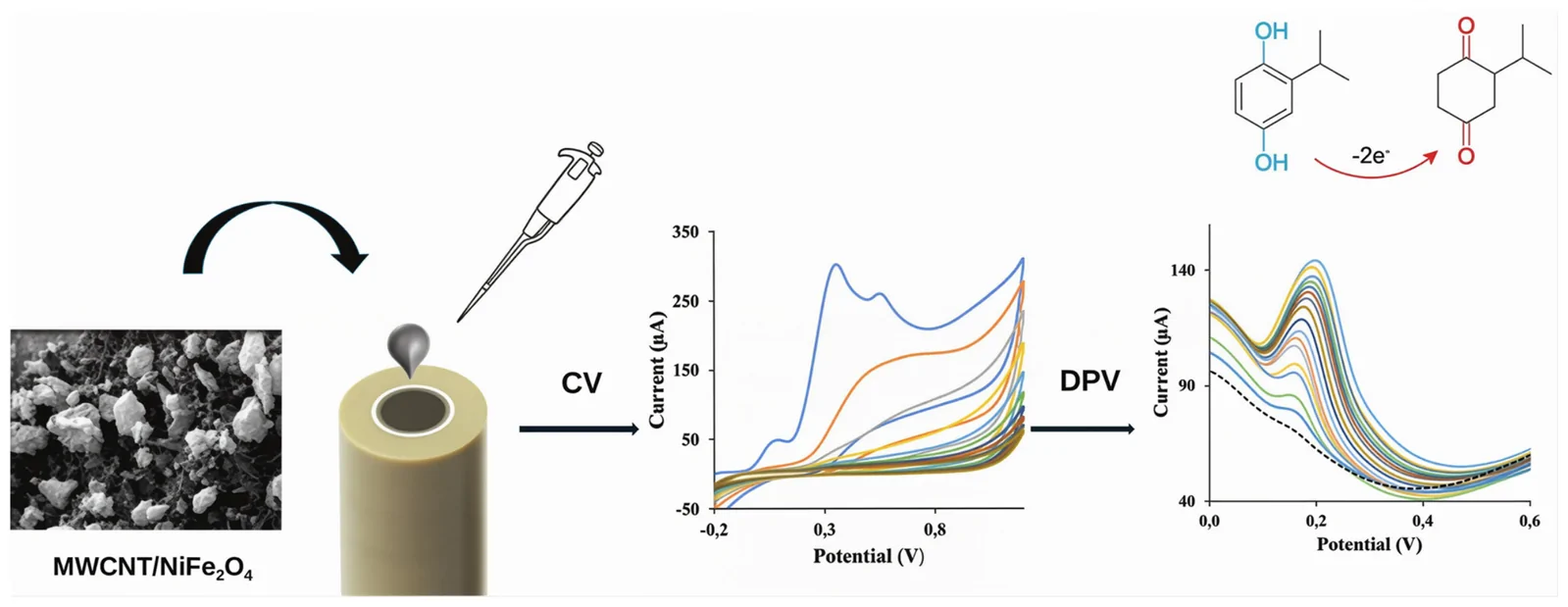
Modification procedure of GCE surface. Different colors represent distinct components and modification steps in the electrode fabrication process. Reproduced with permission from [83].

With the increasing globalization of the food supply chain, the chain from the farm to the table has become longer, and the risk points have increased. The traditional centralized laboratory testing mode is increasingly unable to meet the timeliness requirements. At the same time, the production process of food also needs to strictly control the content of harmful substances. Therefore, on-site rapid detection of antioxidants has emerged. The emerging technologies in this stage include spectroscopy analysis, electrochemical sensing, and biological sensing, etc. These technologies have removed the limitations of large-scale precision instruments and simplified the sample pre-treatment process. The detection time has been reduced from several hours to a few minutes [78,79,80,81,82]. For instance, Soylu et al. [83] developed an electrochemical sensor based on a molecularly imprinted polymer (MIP)-modified NiFe_2_O_4_/multi-walled carbon nanotube (MWCNT) nanocomposite for the detection of tert-butylhydroquinone (TBHQ) in edible oil. By modifying the surface of a glassy carbon electrode with MIP/MWCNT/NiFe_2_O_4_, the conductivity, specific surface area, and active sites were significantly enhanced. Under optimal conditions, the linear range of differential pulse voltammetry for detecting TBHQ was 0.5–25 μM and 25–600 μM, with a detection limit as low as 0.05 μM. This sensor exhibited excellent stability, selectivity, and repeatability, and the spiked recovery rate in actual oil samples was 96.90–104.20%, with RSD lower than 2.94%, providing an effective new method for the rapid detection of TBHQ in edible oil (Figure 1). The new rapid detection technology has enabled the detection to gradually shift from offline precise detection in the laboratory to on-site, real-time and intelligent rapid screening. However, it should be noted that these advantages are context-dependent; spectroscopic methods still face selectivity limitations, while electrochemical sensors require careful evaluation of long-term stability and reproducibility under real-world conditions.

### 3.5. Overview of Sample Pretreatment Techniques

Regardless of the detection technique used, sample preparation is a critical step that affects the accuracy and reproducibility of analytical results. Food matrices have complex compositions; co-existing substances such as fats, proteins, pigments, and carbohydrates not only interfere with the separation of target compounds and their signal responses but may also contaminate chromatographic columns or passivate electrode surfaces. Therefore, sample preparation steps are necessary to extract, purify, and enrich the target antioxidants.

The traditional pre-treatment methods are mainly represented by liquid–liquid extraction and solid-phase extraction. Liquid–liquid extraction extracts the target substances based on the distribution difference between the organic phase and the aqueous phase. It is simple to operate and has low cost, but it consumes a large amount of organic solvents, frequently causes emulsification, and is difficult to handle high lipid matrices [84]. Solid-phase extraction selectively retains the target substances through the adsorbent and then elutes them. Its purification effect is superior to that of liquid–liquid extraction. However, the operation process is cumbersome and time-consuming, and the solid-phase extraction column is a one-time consumable material, resulting in high detection costs [85,86].

In recent years, to meet the demands for rapid detection and high-throughput analysis, miniaturized and green pre-treatment technologies have developed rapidly. Dispersed liquid–liquid microextraction rapidly injects trace extraction solvents and dispersants into the sample solution, forming a cloud-like microemulsion, which greatly increases the contact area between the extraction solvent and the target substance, and can complete the extraction within several minutes. For example, Mogaddam et al. [87] developed a dispersion-liquid–liquid microextraction (DLLME) pretreatment method based on heated liquid–liquid extraction combined with a switchable low-eutectic solvent (DES) for the extraction of three phenolic antioxidants from edible oils. In this method, a trace amount of DES extractant is dissolved in an alkaline solution to form a homogeneous system; upon injection of an acidic solution, an acid-base reaction occurs, causing the target compounds to migrate into the fine DES droplets, thereby completing the extraction. The entire extraction process requires no organic solvents and is simple and cost-effective. Method validation results showed that the limits of detection for the three antioxidants ranged from 0.13 to 0.42 ng/mL, with enrichment factors of 370–445, extraction recoveries of 74–89%, and relative standard deviations ≤ 7.4%. This method was successfully applied to the quantitative analysis of antioxidants in actual edible oil samples.

Overall, the development trend of sample pretreatment techniques is towards miniaturization, automation, low solvent consumption, and online integration. Chromatography has the highest dependence on pretreatment, followed by spectroscopy. Electrochemistry, on the other hand, has relatively less stringent requirements for pretreatment as it can directly detect in complex matrices. Therefore, electrochemistry has significant advantages in field applications, although challenges such as electrode fouling and matrix effects require careful consideration.

### 3.6. Development and Progress of Chromatographic Analysis Technology

Chromatographic analysis is the most mature and widely applied technical method for detecting food antioxidants. The mainstream chromatography techniques are divided into two branches: gas phase and liquid phase. In the field of antioxidant detection, each category has its specific applicable objects and technical characteristics.

The evolution of chromatographic techniques for antioxidant analysis has progressed through several stages: from conventional liquid chromatography (LC) and gas chromatography (GC) for single-compound analysis, to high-performance liquid chromatography (HPLC) with UV or fluorescence detection for multi-component separation, and more recently to ultra-high-performance liquid chromatography (UHPLC) and comprehensive two-dimensional chromatography (LC × LC, GC × GC) coupled with high-resolution mass spectrometry (HRMS) for untargeted profiling and trace-level quantification. The development of reversed-phase HPLC (RP-HPLC) with C18 stationary phases, combined with diode array detection (DAD), enabled simultaneous separation and spectral identification of multiple phenolic antioxidants. The hyphenation of chromatography with mass spectrometry—from GC-MS to LC-MS/MS and HRMS—further enhanced sensitivity and structural elucidation capability, making these techniques indispensable for confirmatory analysis in food safety laboratories.

Liquid chromatography is currently the most widely used technique in antioxidant analysis. It can be further classified based on the separation mechanism into normal-phase high-performance liquid chromatography and reversed-phase liquid chromatography [88]. Polar chromatography is mainly used for separating polar compounds such as phenolic acids and flavonoids that are derived from natural sources [89,90,91]. Reversed-phase high-performance liquid chromatography is the most commonly used method. By using a C18 column in combination with a mobile phase system of acetonitrile-water or methanol-water, it can simultaneously separate BHA, BHT, TBHQ, PG, and a variety of natural phenolic antioxidants within one analytical cycle [92,93]. For example, Casagrande et al. [94] developed a reverse-phase liquid chromatography method for the direct analysis of four synthetic phenolic antioxidants (PG, TBHQ, BHA, and BHT) in biodiesel. Although biodiesel is not a food matrix, this study provides valuable methodological insights into the separation of phenolic antioxidants from lipid-rich matrices using a phenyl column via π-π interactions, which are transferable to edible oil analysis. The study found that BHT co-eluted with fatty acid methyl esters on a C18 column, whereas an alkylphenyl column achieved good separation of the antioxidants from the matrix through π-π interactions. This method exhibits good linearity in the range of 10–80 ppm (R^2^ > 0.9986), with detection limits of 0.010–0.030 ppm and spiked recovery rates of 70–103%.

**Figure 2:**
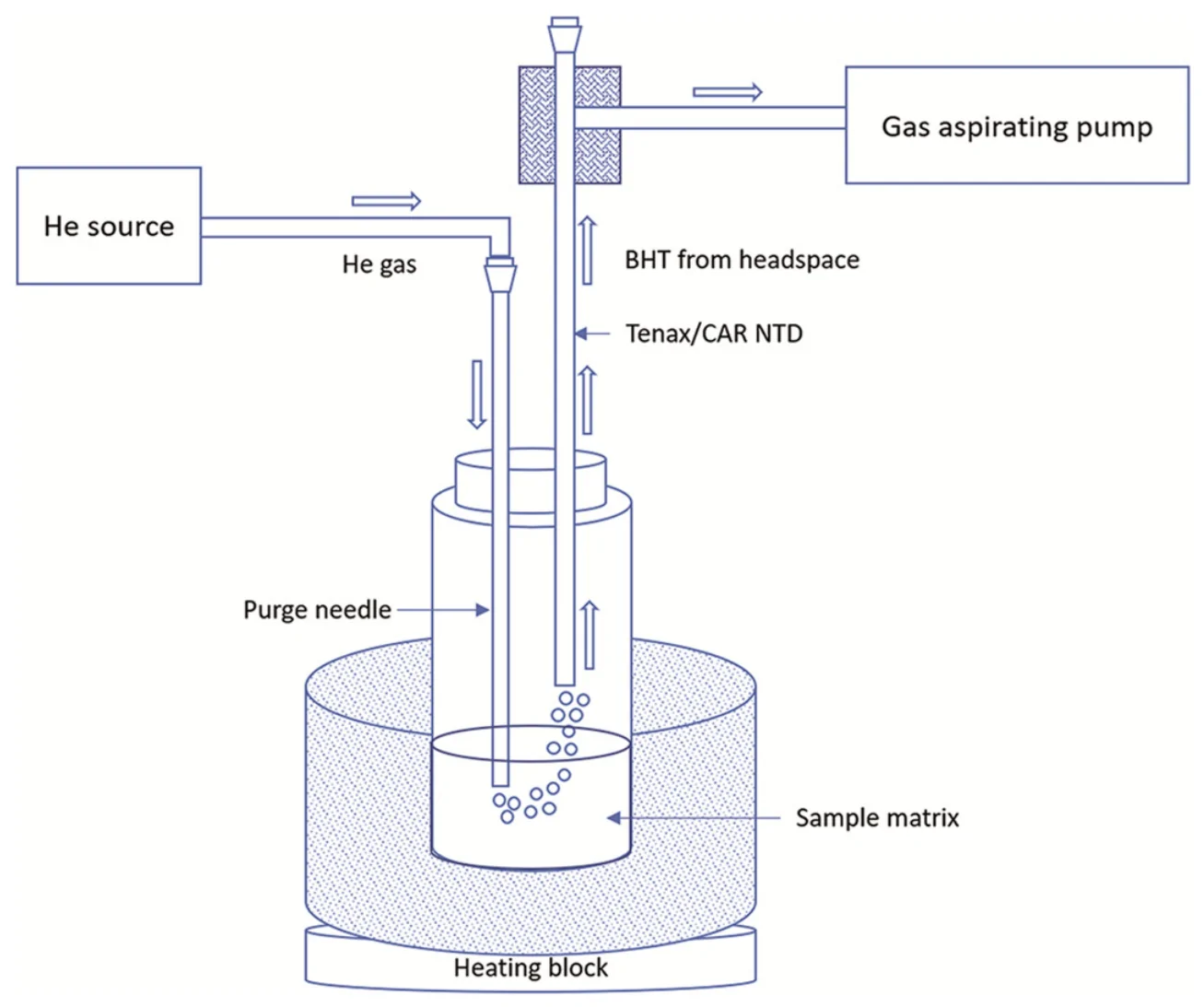
Schematic illustration of the experimental setup for BHT extraction from headspace using a Tenax/CAR (Carboxen) NTD (needle trap device) coupled with a heating block and gas aspiration. Reproduced with permission from [101].

Gas chromatography utilizes the difference in distribution between the sample components in the gaseous state and the stationary phase of the chromatographic column to achieve separation. It is suitable for those antioxidants with strong volatility and good thermal stability [95,96,97,98]. BHA and BHT are the most commonly analyzed compounds in gas chromatography. These two synthetic phenolic antioxidants have low molecular weights and moderate boiling points; they vaporize readily at an injection port temperature of around 200 °C without undergoing thermal degradation, allowing them to be analyzed directly by gas chromatography without the need for complex derivatization pretreatment. For food matrix applications, Znidersic et al. [99] developed an SPME-GC-MS/MS method for the simultaneous determination of nine compounds (including phenolic antioxidants, parabens, plasticizers, and flame retardants) expected to migrate from food contact materials into beverages and vinegar, with no derivatization required. The method achieved detection limits of 0.005–0.2 μg/L, mean recoveries of 98–109%, and RSD values of 0.8–5.4%. Similarly, Chen et al. [100] established a headspace solid-phase microextraction (HS-SPME) coupled with GC-MS method for the analysis of synthetic phenolic antioxidants in food-grade lubricant samples, demonstrating recoveries of 80.7–109.6%. These studies confirm the applicability of GC-MS for volatile antioxidant analysis in food-related matrices. Additionally, Ghosh et al. [101] demonstrated the utility of a headspace needle trap combined with portable GC-MS for rapid BHT quantification in cosmetics, highlighting the potential of portable GC-MS for on-site screening applications (Figure 2). For phenolic compounds with high boiling points or those prone to decomposition upon heating—such as TBHQ and gallate esters—the application of gas chromatography is limited, often requiring derivatization to reduce polarity and improve thermal stability, which adds to the number of steps and increases analysis time. For example, Xu et al. [102] successfully determined TBHQ and its metabolites in fats and oils simultaneously using gas chromatography–mass spectrometry (GC-MS). GC generally delivers higher separation efficiency and faster analysis than HPLC, yet sample-preparation workflows are more complex and demand higher operator proficiency. In addition, thermally unstable compounds may decompose during vaporization, limiting the application of GC in the detection of certain phenolic antioxidants.

Beyond conventional HPLC and GC, ultra-high performance liquid chromatography (UHPLC) with sub-2 μm particles has further improved separation efficiency and reduced analysis time. Liquid chromatography-tandem mass spectrometry (LC-MS/MS) has become increasingly important for antioxidant analysis, offering superior sensitivity, structural confirmation capability, and the ability to simultaneously quantify multiple targets in complex food matrices [103,104,105]. This is particularly advantageous for thermally labile and polar phenolic antioxidants that are not amenable to GC analysis.

In summary, the choice between GC and LC depends primarily on the physicochemical properties of the target antioxidants: GC is suitable for volatile and thermally stable compounds such as BHA and BHT, often with minimal derivatization; LC is preferred for polar, non-volatile, or thermally labile compounds such as TBHQ, PG, and natural polyphenols, and is more compatible with high-sensitivity mass spectrometric detection. Derivatization is generally required for GC analysis of phenolic antioxidants containing multiple hydroxyl groups to reduce polarity and improve thermal stability.

### 3.7. Development and Progress of Spectroscopic Analysis Technology

The mainstream spectroscopic techniques for food antioxidant detection include ultraviolet-visible absorption spectroscopy, molecular fluorescence spectroscopy, Fourier transform infrared spectroscopy, and surface-enhanced Raman spectroscopy (SERS) [36,106,107]. The application of spectroscopic techniques for antioxidant analysis has progressed from conventional UV-Vis absorption and fluorescence methods to more advanced techniques such as SERS, enabled by the development of nanostructured substrates and chemometric algorithms. They achieve the detection by relying on the optical characteristic signals of the phenolic substances themselves. Unlike chromatography, which requires a complex separation process, the sample pretreatment is relatively simple and analysis proceeds rapidly.

**Figure 3:**
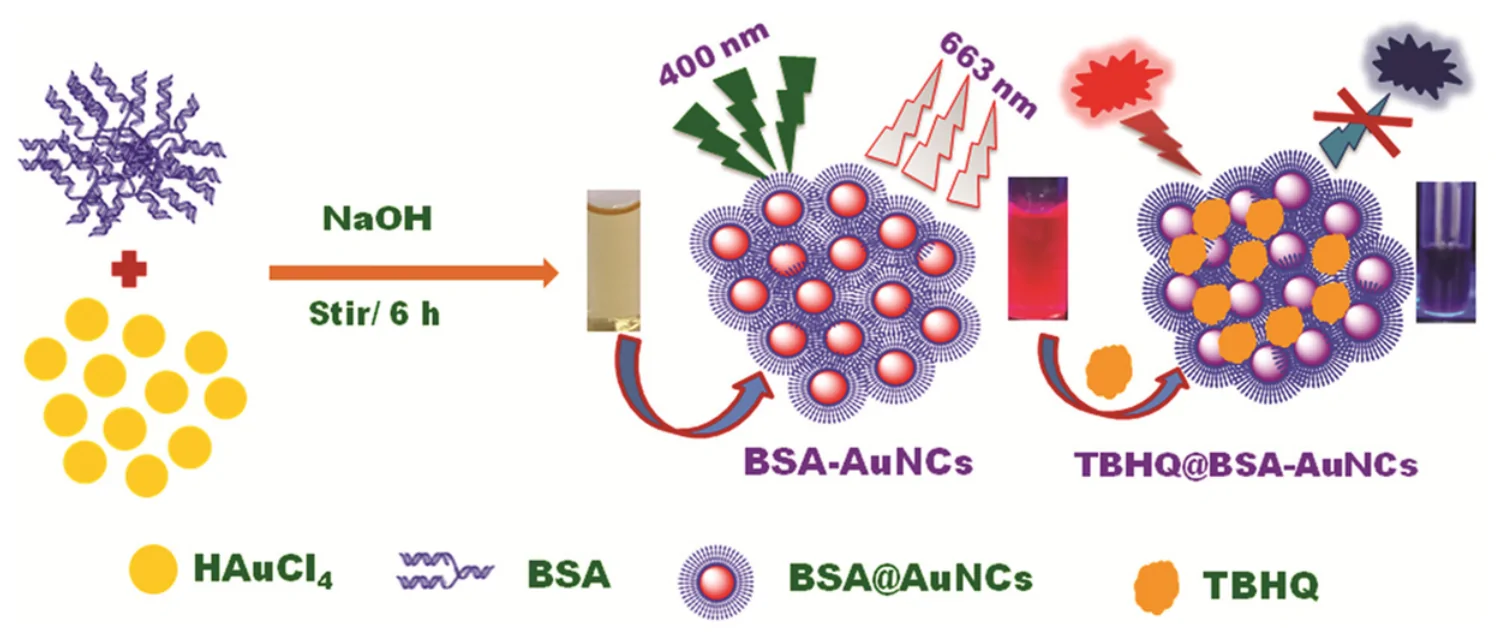
Schematic diagram of the BSA-AuNCs-based fluorescence sensor for TBHQ detection. Reproduced with permission from [108].

Compared to surface-enhanced Raman spectroscopy, UV-visible absorption spectroscopy, Fourier transform infrared spectroscopy, and molecular fluorescence spectroscopy exhibit distinct application boundaries for antioxidant analysis. UV-visible absorption spectroscopy is typically combined with various colorimetric reaction systems to evaluate the overall antioxidant strength or total phenolic content of a sample, among other parameters, yielding a comprehensive numerical value. Molecular fluorescence spectroscopy mostly relies on the quenching or enhancement effects of antioxidants on fluorescent probes to indirectly reflect their activity levels, providing information on total quantity or total activity; it lacks the ability to identify individual substances in multicomponent mixtures. However, if highly selective probes are designed, sensitive detection of a specific single target can be achieved. For instance, Shankar et al. [108] developed a dual-mode (fluorescence/colorimetric) sensing method for detecting the food antioxidant TBHQ based on gold nanoclusters coated with bovine serum albumin (BSA-AuNCs). TBHQ quenches the red fluorescence of the gold nanoclusters through electrostatic interaction. This method has high selectivity for TBHQ, and common phenolic analogues do not interfere. The detection limit is as low as 72 nM, and it has been successfully applied to the detection of TBHQ in edible oil samples (Figure 3). The Fourier transform infrared spectroscopy relies on the superimposition of O–H stretching vibrations in phenolic compounds, C=C vibrations in aromatic rings, and absorption peaks of oxygen-containing functional groups to form an infrared fingerprint spectrum. By comparing it with the standard sample spectrum or using chemometric methods such as principal component analysis, the type identification of the sample can be completed. FTIR coupled with chemometric algorithms enables reliable quantitative analysis; however, its quantitative performance is readily compromised by severe food-matrix interference, restricting its practical application for trace-level target analysis.

**Figure 4:**
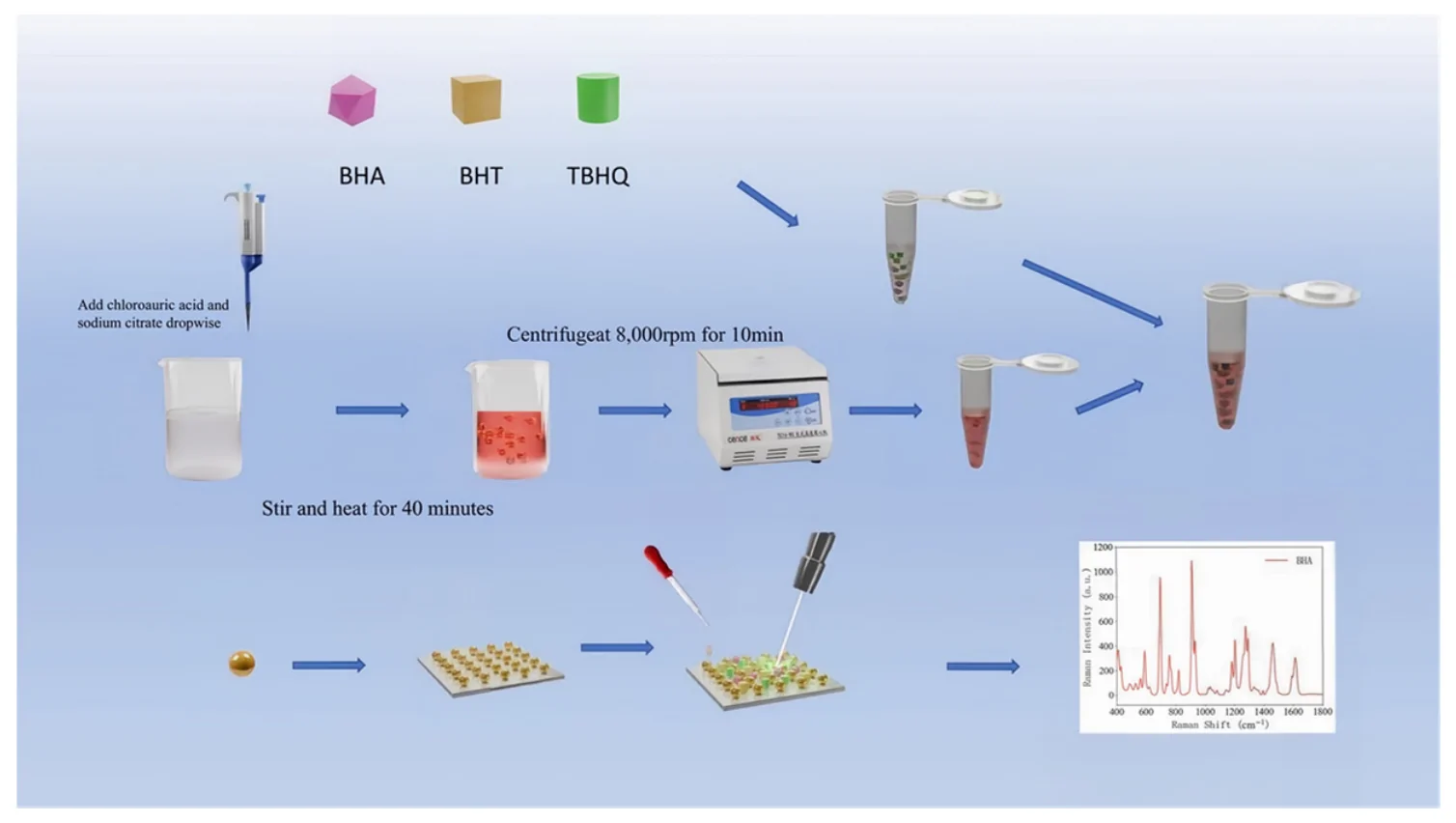
Schematic illustration of the SERS-based detection of BHA, BHT, and TBHQ using gold nanoparticles. Reproduced with permission from [119].

Among these spectroscopic techniques, SERS is particularly attractive for antioxidant detection because its Raman scattering peaks correspond to molecular vibrational modes, providing unique fingerprint spectra for individual phenolic antioxidants. Unlike UV-Vis absorption, conventional fluorescence, and FTIR spectroscopy—which are more commonly employed for total phenolic content (TPC) or total antioxidant capacity (TAC) assessment—SERS enables the identification and quantification of specific antioxidant compounds in complex matrices [109,110,111,112,113]. Unlike the other three types of spectroscopic techniques, the Raman scattering peaks correspond to the vibration modes of chemical bonds within molecules. Each phenolic antioxidant has its own unique Raman fingerprint spectrum. Surface-enhanced Raman spectroscopy technology has made certain progress in antioxidant detection. It can achieve a relatively low detection limit for synthetic antioxidants such as BHA and TBHQ, and the detection time has been reduced from several tens of minutes in chromatography to a few minutes or even shorter. The sample pretreatment is also much simpler than chromatography [114,115,116,117]. Pan et al. [118] developed a quantitative detection method for tert-butylhydroquinone (TBHQ) in plant oils by combining surface-enhanced Raman spectroscopy with chemometrics. The minimum detection concentration of this method in the standard solution was 5 mg/L, and the detection limit in plant oils was 10 mg/kg. A calibration model was established using partial least squares and support vector machine regression, and the predicted values of TBHQ content in actual oil samples were highly correlated with the true values. This provided a new idea for the rapid detection of antioxidants in complex food matrices. Wang et al. [119] used gold nanoparticles (AuNPs) as the active substrate for surface-enhanced Raman spectroscopy (SERS) and combined machine learning algorithms to establish a rapid detection method for three phenolic antioxidants (BHA, BHT, and TBHQ) in edible oils. The enhancement factor of the SERS substrate for rhodamine 6G was 4.4 × 10^5^. The three antioxidants showed good concentration responses in the range of 20–1000 mg/kg, and the detection limit was lower than 10 mg/kg. On this basis, a classification model was constructed using CNN (convolutional neural network) and other machine learning algorithms, and the recognition accuracy for six addition scenarios (single and binary mixtures) reached 99.54%; further, PLS-DA (partial least squares discriminant analysis) was used for over-limit discrimination analysis, with an accuracy rate exceeding 94%. This study provided a new idea for on-site and rapid SERS screening of antioxidants in edible oils (Figure 4).

### 3.8. Development Progress of Electrochemical Detection Technology

Electrochemical analysis is a quantitative detection technique based on the current, potential or impedance signals generated when the analyte undergoes oxidation-reduction reactions at the electrode surface. The content of the analyte is determined by measuring the intensity of the electrical signal [120,121,122,123]. Compared with chromatography and spectroscopy, electrochemical detection has advantages in terms of instrument cost, response speed, operational convenience and miniaturization potential. Due to the continuous development of modified electrode materials, detection performance has been significantly improved. The evolution of electrochemical detection can be divided into four stages based on electrode modification strategies.

Bare Electrode Stage. Early studies directly used glassy carbon, gold, or carbon paste electrodes for phenolic antioxidant detection. These bare electrodes generally deliver only micromolar-level limits of detection. Moreover, co-existing electroactive interferents (e.g., ascorbic acid, uric acid, dopamine) with similar oxidation potentials produce overlapping signals, resulting in poor selectivity [124,125]. Electrode passivation induced by oxidation-product accumulation further degrades reproducibility [80]. Surface modification using nanomaterials provides an effective route to overcome these intrinsic drawbacks and improve overall sensing performance.

**Figure 5:**
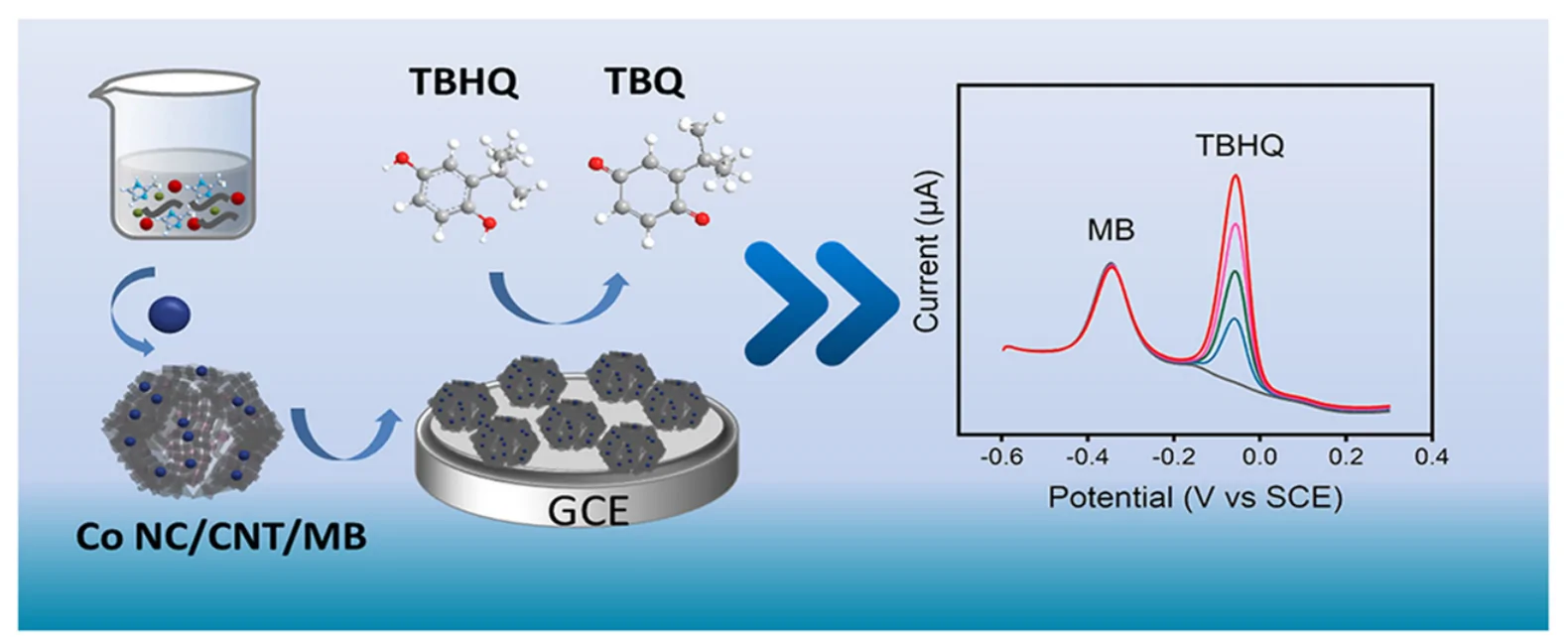
Graphical abstract of a ratiometric electrochemical sensor for TBHQ detection constructed using Co NC/CNT composites and methylene blue (MB) as an internal standard. Reproduced with permission from [132].

Carbonaceous and Metallic Nanomaterial Modification. The introduction of carbon-based nanomaterials (carbon nanotubes, graphene, carbon black) and noble metal nanoparticles (AuNPs, AgNPs, PtNPs) significantly enhanced electrode conductivity, surface area, and catalytic activity, substantially improving detection sensitivity [126,127,128,129,130,131]. Representative examples include a ratiometric sensor based on Co/N-doped carbon nanotube composites for TBHQ detection (LOD: 0.054 μM) [132], and a CB@Au/poly (p-ABSA) nanocomposite sensor achieving a 10 nM LOD for TBHQ in edible oil [14] (Figure 5).

**Figure 6:**
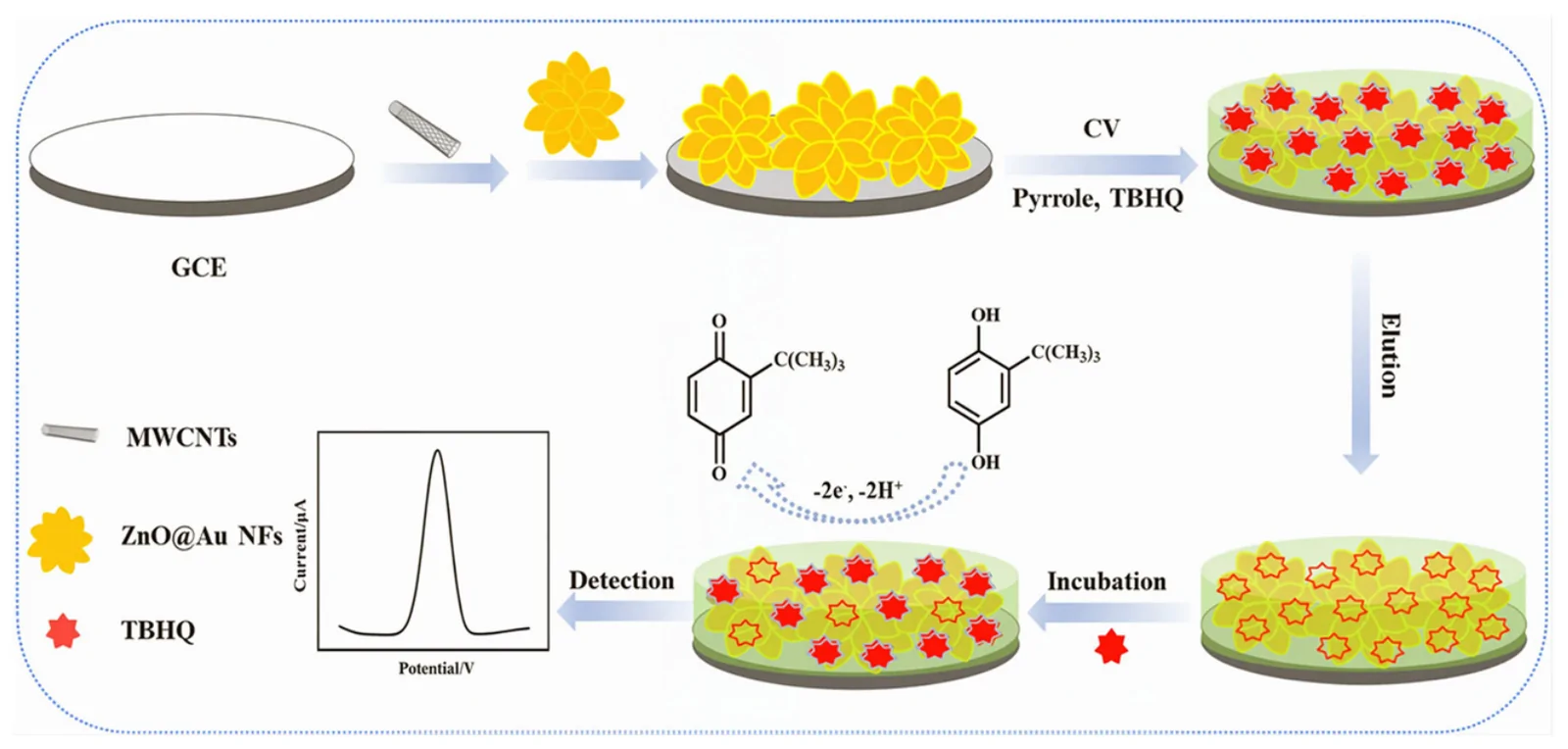
Schematic illustration of the construction principle and redox mechanism of a molecularly imprinted electrochemical sensor based on MWCNTs and flower-like ZnO@Au NFs for TBHQ detection. Reproduced with permission from [138].

Functional Porous Material Modification. The introduction of MXenes, metal-organic frameworks (MOFs), covalent organic frameworks (COFs), and metal oxide/sulfide nanomaterials further enhanced sensing performance [133,134,135,136]. These materials offer high porosity, large surface areas, ordered pore structures, and abundant active sites, which facilitate analyte enrichment and improve sensitivity. For example, Srinivasan et al. [137] constructed a Mn-MOF@f-CNF/SPE sensor for TBHQ detection, achieving a linear range of 0.01–800 μM with a 1.36 nM LOD, and successfully applied it to potato chips and instant noodles. Han et al. [138] developed a ZnO@Au flower-like nanocomposite with a molecularly imprinted polymer for TBHQ detection, exhibiting a linear range of 0.5–200 μmol/L and a 58 nmol/L LOD (Figure 6).

**Figure 7:**
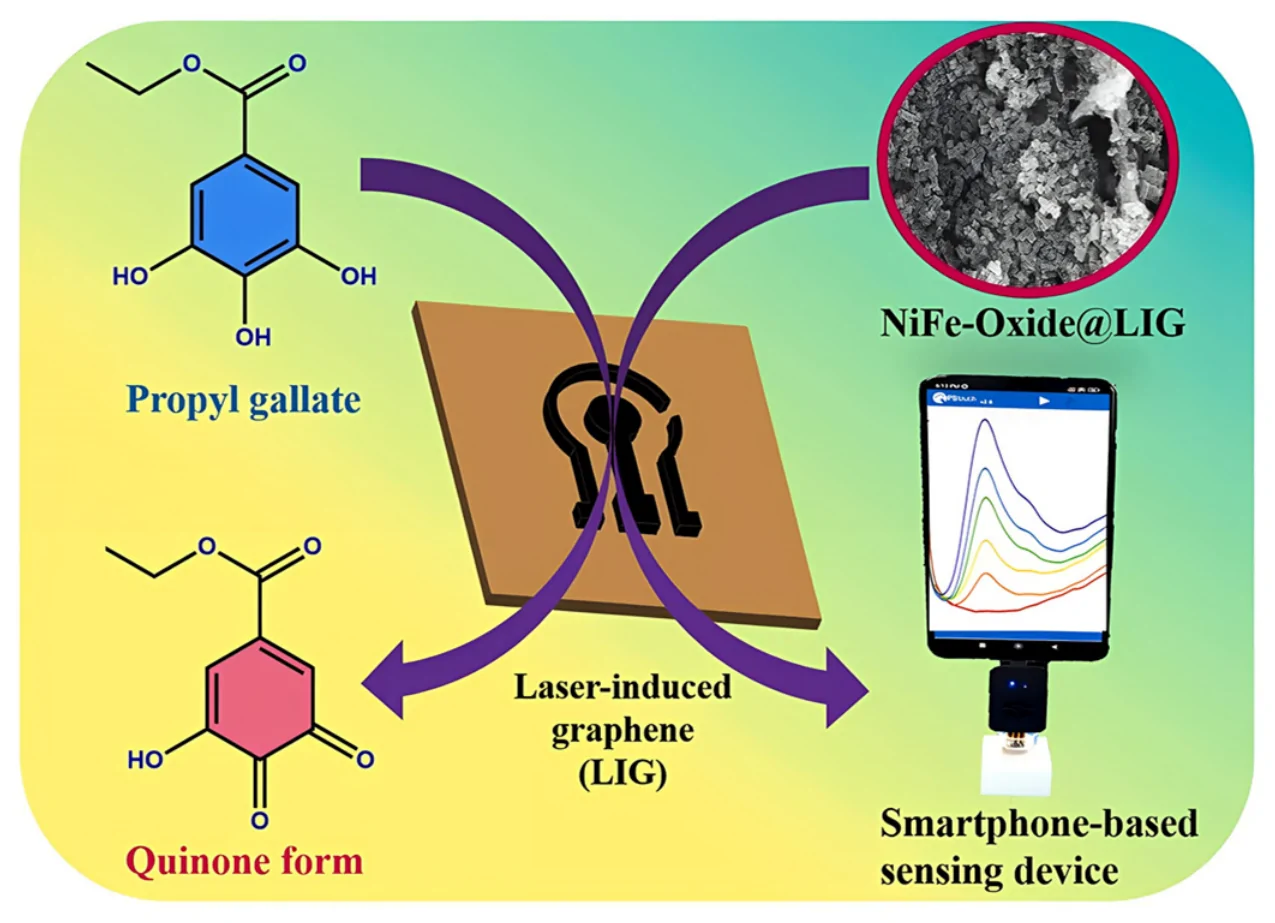
Schematic diagram of a smartphone-based electrochemical sensing method for propyl gallate in food samples using NiFe-oxide-decorated flexible laser-induced graphene electrodes. Reproduced with permission from [139].

Integrated Intelligent Sensing. By integrating microfluidic channels, flexible conductive substrates, and smartphone signal acquisition modules, integrated sensing platforms have been developed for on-site and real-time monitoring. Chinnapaiyan et al. [139] fabricated a flexible laser-induced graphene (LIG) electrode modified with porous NiFe oxide for smartphone-based electrochemical detection of PG in edible oils, achieving a 0.01 μM LOD (Figure 7).

**Figure 8:**
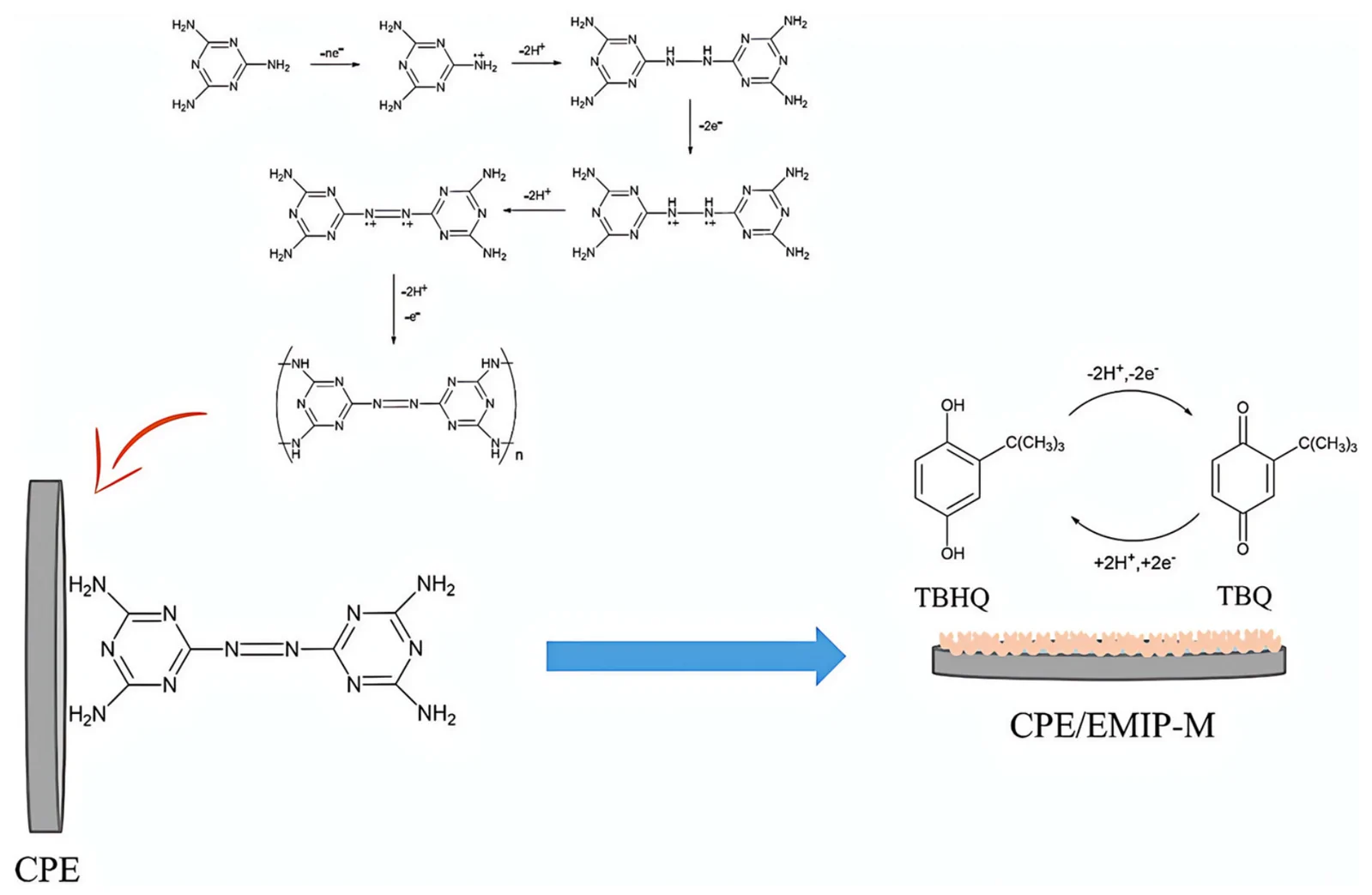
Schematic illustration of the CPE/EMIP-M sensor constructed via in situ electropolymerization of melamine and molecular imprinting technology for the electrochemical detection of TBHQ. Reproduced with permission from [143].

Despite these advances, selectivity remains a key challenge for electrochemical sensing in food applications. Common electroactive endogenous compounds (ascorbic acid, dopamine, uric acid) can produce overlapping signals, while polymerization and adsorption of phenolic oxidation products may cause electrode fouling. To address these limitations, molecularly imprinted polymers (MIPs) and metal–organic frameworks (MOFs) have been introduced to provide specific recognition cavities [140,141,142]. Noviandri et al. [143] used melamine as the functional monomer and TBHQ as the template molecule to prepare a molecularly imprinted polymer (MIP)-modified electrode through an electrochemical polymerization method on the surface of a carbon paste electrode. This electrode was used for the square wave voltammetric detection of TBHQ. The electrochemical molecularly imprinted polymer (EMIP-M) provided a specific recognition cavity, endowing the sensor with good selectivity. Under optimized conditions, the linear detection range of this sensor was 1–1000 μM, and the detection limit was 0.29 μM. The detection results in actual food samples showed no significant difference from those obtained by the HPLC method (Figure 8).

By analyzing the development progress of various technologies, it can be observed that the chromatography, spectroscopy, and electrochemistry detection techniques have been continuously evolving and upgrading along the directions of precise separation, rapid screening, and in situ online detection. Currently, none of these technologies can completely replace the other two types [144,145,146]. This also indicates that in actual testing work, multiple factors such as the characteristics of the sample to be tested, the detection sensitivity standards, and the on-site operational conditions need to be considered. Appropriate detection methods should be selected flexibly. In practical applications, the three techniques do not operate independently, but have formed a good complementary system. Among them, Chromatography delivers outstanding separation performance and can provide precise benchmark verification basis for spectral and electrochemical detection results; spectral technology has a fast response speed and is more suitable for the preliminary screening of large quantities of samples; electrochemical technology has a simple equipment structure and is easy to be miniaturized, effectively compensating for the technical shortcomings of real-time rapid detection in the field. Through multi-dimensional comparative analysis of the three types of detection technologies (Table 2), this article can provide reliable references for practical method selection.

## 4. Summary and Prospect

This article systematically reviews the classification system of phenolic antioxidants in food and the latest research progress in their detection techniques, with a focus on evaluating the advantages and limitations of the three major mainstream technical paths: chromatographic analysis, spectroscopic analysis, and electrochemical detection. Due to significant differences in sources, solubility, and chemical structure, the detection methods for food antioxidants have evolved from laboratory chemical titration, precise instrumental analysis to on-site rapid screening. Currently, chromatographic technology, with its outstanding separation ability and excellent quantitative accuracy, remains the gold standard for laboratory confirmatory analysis; spectroscopic technology (especially SERS) is suitable for initial screening of large quantities of samples due to its simple operation and rapid response; while electrochemical sensing technology demonstrates unique potential in on-site real-time detection and wearable monitoring scenarios due to its low equipment cost, high sensitivity, and ease of miniaturization. Although all these methods have made significant progress, no single technology can simultaneously meet the combined requirements of high sensitivity, high selectivity, rapid response, and simultaneous detection of multiple components. Chromatography is limited by expensive equipment, complex pre-treatment, and large consumption of organic solvents; spectroscopy is susceptible to interference from food matrices and has insufficient selectivity; electrochemical sensing faces bottlenecks such as electrode contamination and interference from electroactive substances. Therefore, in practical applications, it is necessary to flexibly select or combine multiple technologies based on the detection purpose, sample characteristics, and on-site conditions to form a complementary detection strategy.

The development of future food antioxidant detection technologies should focus on the deep integration and systematic integration of multiple technical paths to achieve full coverage from laboratory precise analysis to on-site real-time monitoring. By fully leveraging the complementary advantages of chromatography in separation and confirmation, spectroscopic methods in rapid screening, and electrochemical sensing in portable detection, it is expected to construct a more flexible and adaptable detection system, satisfying differentiated requirements for diverse food matrices and application scenarios. At the same time, continuous innovation in new nanomaterials, porous framework materials, and molecular imprinting polymers will provide strong support for improving sensor selectivity and enrichment efficiency, promoting further improvement in detection performance. Future research should also focus on establishing a comprehensive antioxidant detection method database, systematically tracking the applicability and impact of different detection strategies on various food matrices and target substances, and developing efficient mixed-association strategies to improve the detection sensitivity and selectivity in complex systems. Beyond technological innovation, translating these methods into practice also demands rigorous validation in real food matrices, standardized protocols for inter-laboratory reproducibility, and regulatory acceptance supported by thorough performance data. Closing the gap between academic development and industrial adoption remains a key challenge. These advancements will drive the development of fields such as food safety regulation, on-site rapid screening, online real-time quality control, and green analytical chemistry, ensuring that detection technologies continue to play a foundational role in ensuring food quality and consumer health.

## Figures and Tables

**Table 2:** Comparison of antioxidant detection methods.

Comparison Aspects	Chromatographic Technique	Spectroscopic Technology	Electrochemical Detection
Analytic target	Qualitative and quantitative analysis of individual target components	Total antioxidant capacity, or the quantitative measurement of specific known components	Total antioxidant capacity, as well as the quantification of specific active components
Analysis index	Retention time (qualitative), peak area/peak height (quantitative)	Absorbance, fluorescence intensity, chemiluminescence intensity	Oxidation peak potential (qualitative), peak current (quantitative)
Key Advantages	Excellent separation capability, with accurate qualitative and quantitative results	The operation is simple, fast, and the cost of the reagents is low.	No complex preprocessing is required. the response is fast and the sensitivity is high.
Main limitations	The instrument is expensive, requires professional training for operation, has a long analysis time, and needs a large amount of organic solvents.	Low selectivity, prone to interference, and poor comparability	The electrode fouling and does not respond to non-electroactive antioxidants.
Sample treatment	Complex, both extraction and solid-phase purification are indispensable.	Simple. some can be directly diluted before injection.	Relatively simple. the Single-use electrodes do not require complex purification.
Selectivity	Extremely high	Medium (with only SERS/fluorescence probes showing strong specificity)	Medium (the MIP modification can significantly enhance it)
Duration of single-sample testing	10–60 min	1–5 min	0.5–3 min

## Data Availability

No new data were created or analyzed in this study. Data sharing is not applicable to this article.
